# Acquired Submitral Aneurysm in an Adolescent with Relapse of B-cell Acute Lymphoblastic Leukemia and History of Multiple Systemic Infections: A Case Report

**DOI:** 10.1007/s00246-025-03947-w

**Published:** 2025-07-11

**Authors:** Colin J. Crilly, Karen Carvalho, David S. Winlaw, Matthew Cornicelli

**Affiliations:** 1 Division of Cardiology, Department of Pediatrics, Ann & Robert H. Lurie Children’s Hospital of Chicago, Northwestern University Feinberg School of Medicine, Chicago, IL USA; 2 Department of Cardiothoracic Surgery, Ann and Robert H. Lurie Children’s Hospital of Chicago, Northwestern University Feinberg School of Medicine, Chicago, IL USA

**Keywords:** Ventricular aneurysm, Hematologic malignancy, Annular rupture, Infective endocarditis

## Abstract

We present the case of a 14-year-old boy with history of B-cell acute lymphoblastic leukemia (ALL), multiple polymicrobial infections, and recent pericardial effusion requiring pericardiocentesis who was incidentally found to have a submitral left ventricular aneurysm. Concurrently, he developed a relapse of his B-cell ALL as demonstrated by the presence of lymphoblasts in his cerebrospinal fluid. The decision was made to proceed with surgical repair of the aneurysm prior to initiation of systemic chemotherapy. The patient underwent a successful repair of the aneurysm through repair of the mitral valve annular disruption. The aneurysm is likely to be due to partially treated endocarditis, weakening the mitral valve atrioventricular junction, resulting in dehiscence. Recovery post-operatively was uneventful and he was able to undergo CAR-T cell therapy several months later.

## Case Report

A 14-year-old boy presented to the Oncology clinic for follow-up of B-cell acute lymphoblastic leukemia (ALL). Initial diagnosis was 10 months earlier. Clinical course was subsequently complicated by invasive mucormycosis with orbital and intracranial extension requiring a craniotomy and left frontal lobectomy, multiple episodes of polymicrobial sinusitis, a polymicrobial intracranial abscess, and methicillin-sensitive staphylococcus aureus (MSSA) bacteremia. The patient was last hospitalized for a large pericardial effusion requiring pericardiocentesis and was seen four days after his discharge. Echocardiography five days prior showed normal biventricular systolic function, mild mitral and aortic valve regurgitation, and a trivial pericardial effusion.

**Fig. 1:**
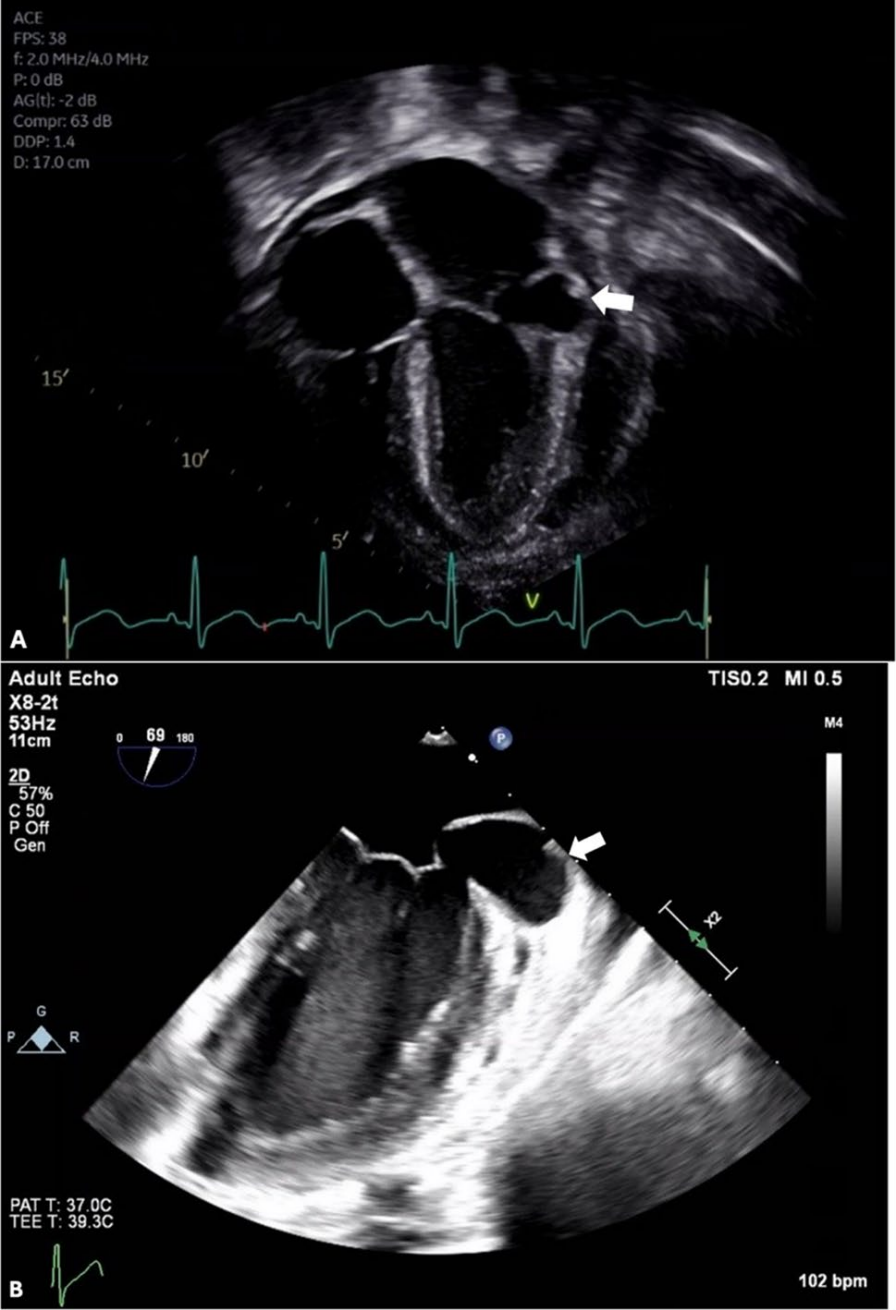
**a** Transthoracic echocardiogram demonstrating an aneurysm located underneath the posterior leaflet of the mitral valve near the mitral valve atrioventricular junction (arrow) **b** Transesophageal echocardiogram at the mid-esophageal two-chamber view demonstrating the 3.9 × 1.8 cm aneurysm as a result of dehiscence in the mitral valve atrioventricular junction fibrosus at the hinge point (arrow)

In the Oncology clinic, the young man was asymptomatic and there were no new infectious concerns. Physical examination was notable for a previously unappreciated 1–2/6 low-pitched pansystolic murmur, best heard at the left lower sternal border. Echocardiography showed a small, globally distributed pericardial effusion and mild mitral valve regurgitation. In addition, there was an aneurysm located underneath the posterior leaflet of the mitral valve, just below the mitral valve annulus. The aneurysm measured 1.7 × 2.1 × 3.1 cm (Fig. 1a).

**Fig. 2:**
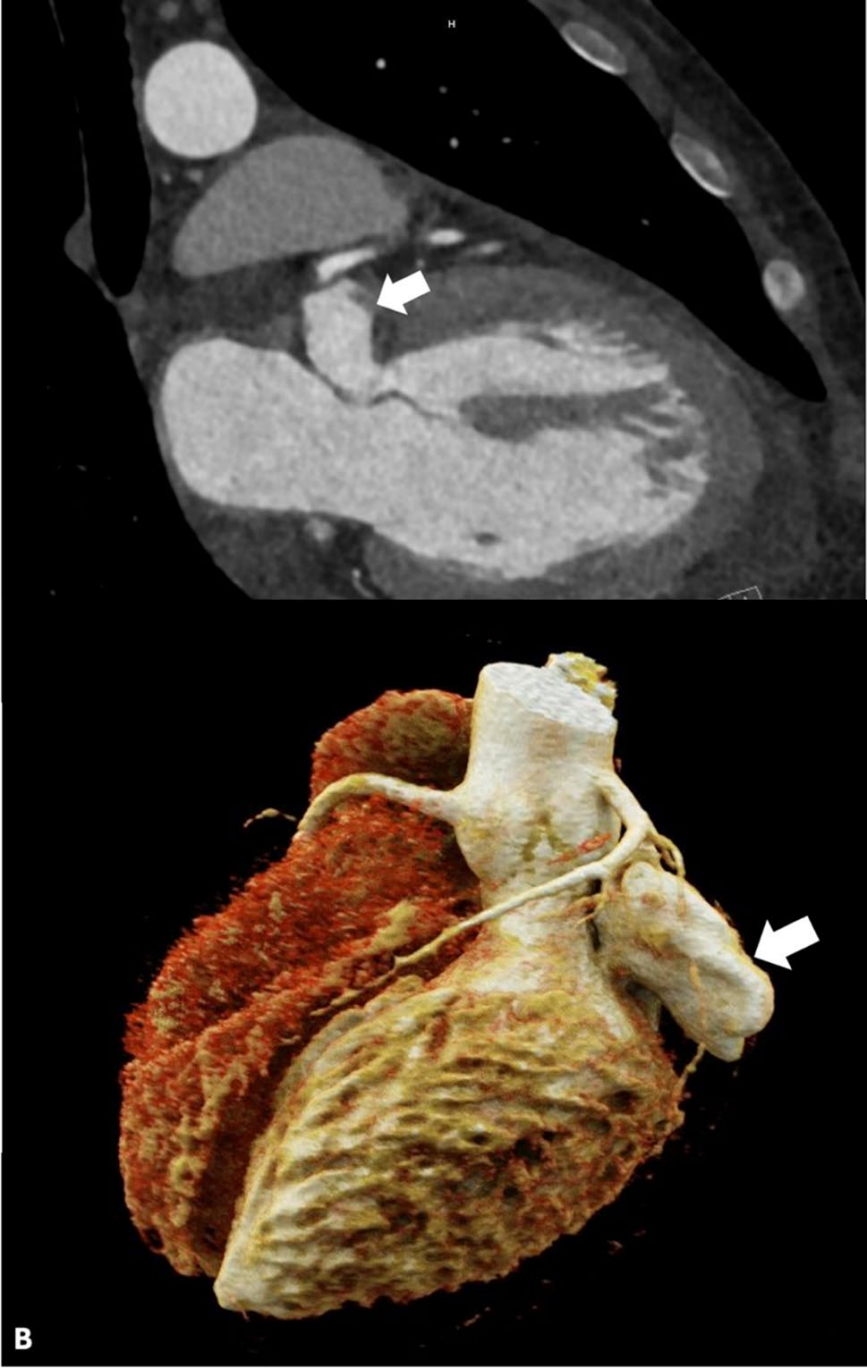
**a** CT imaging demonstrating the submitral aneurysm at the hinge point of the mitral valve (arrow) **b** CT 3D reconstruction demonstrating the aneurysm as it appears from the exterior, in close proximity to the left circumflex coronary artery (arrow)

The patient was directly hospitalized from the out-patient clinic for further cardiac evaluation. Transesophageal echocardiography showed that the aneurysm (3.9 × 1.8 cm in dimension) was caused by a dehiscence in the mitral valve annulus fibrosus at the hinge point just posterior to the left atrial appendage and anterior to the A1 scallop (Fig. 1b). There was to and fro flow between the aneurysm and the left ventricle. Mild mitral valve regurgitation was noted, originating at the A1P1 and A2P2 leaflets as a result of mitral annular ring deformation. In addition, the study noted mild aortic valve regurgitation and mildly depressed left ventricular systolic function with a shortening fraction of 22%. He also underwent a computed tomography scan with angiography of the chest the following day, which redemonstrated the submitral aneurysm with superior displacement and compression upon the left atrial appendage, in close proximity to the left circumflex coronary artery (Fig. 2).

Concurrently, a diagnostic lumbar puncture was performed, which demonstrated lymphoblasts in his cerebrospinal fluid consistent with relapse of the B-cell ALL in the central nervous system. There was no evidence of oncologic disease on a subsequent bone marrow biopsy. The patient therefore received intrathecal chemotherapy during this inpatient stay. Clinical history and cardiac imaging were reviewed at a multidisciplinary conference that included Cardiology, Cardiac Surgery, and Oncology. Ultimately, the decision was made to proceed with repair of the aneurysm with a subsequent waiting period of at least one month before initiation of systemic chemotherapy.

**Fig. 3:**
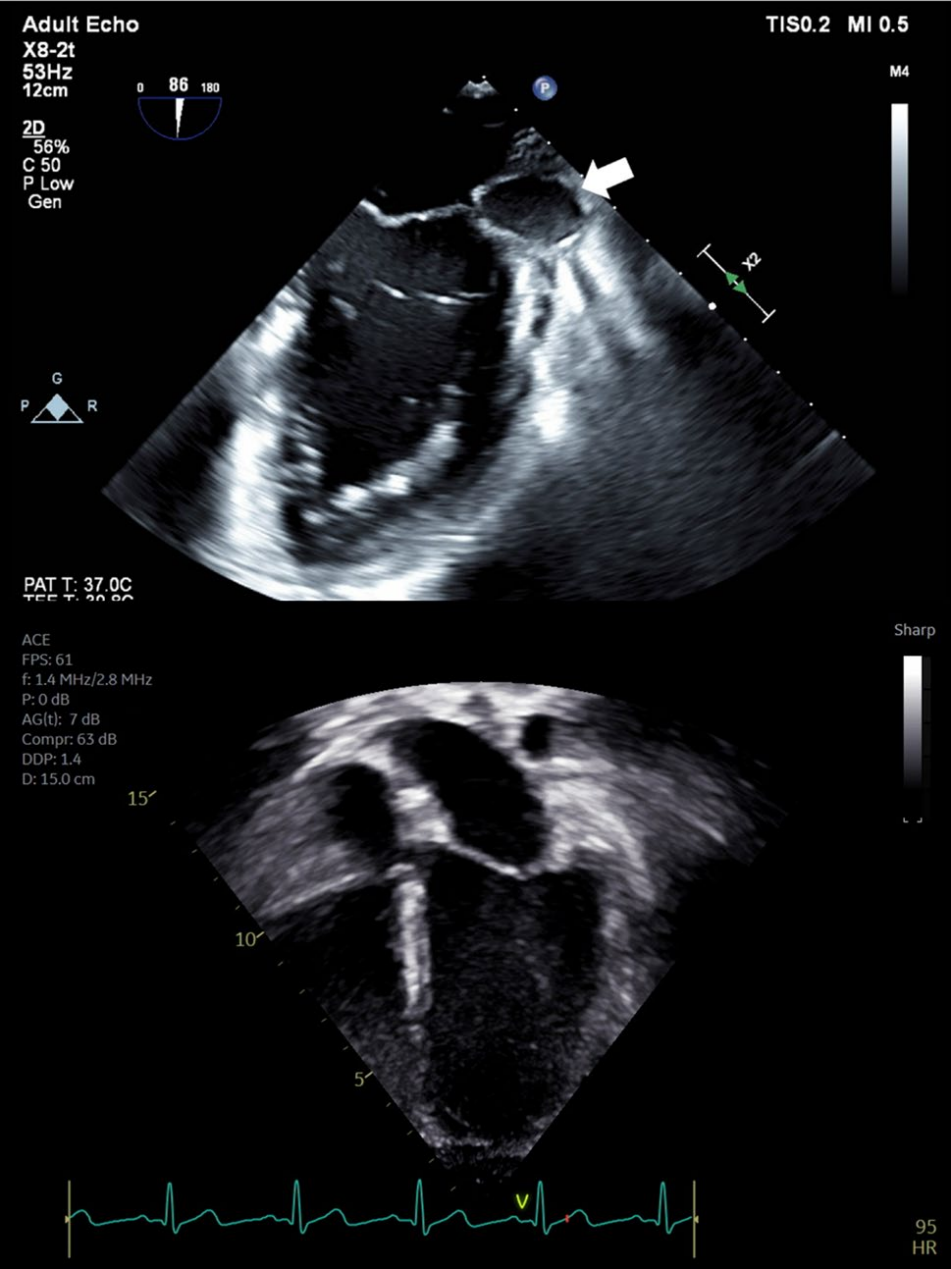
**a** Post-operative transesophageal echocardiogram at the mid-esophageal two-chamber view redemonstrating the submitral aneurysm, now without direct communication with the left ventricle following placement of a bovine pericardial patch (arrow) **b** Transthoracic echocardiogram one month post-surgery showing no further evidence of the submitral aneurysm

In the operating field, a sanguinous effusion in the pericardial sac was identified. On exposing the mitral valve through the left atrium, annular disruption below the P1 component of the posterior mitral leaflet was identified, with direct communication between the ventricle and the thin-walled aneurysm. There was discolored tissue surrounding the disrupted site that, given the high suspicion for infection, was excised and sent for culture. Subsequent pathologic evaluation showed that the tissue had intimal hyperplasia with no identified microorganisms. The area of dehiscence was covered with a bovine pericardial patch and the posterior mitral valve was reattached to the patch at the atrioventricular junction. Post-operative transesophageal echocardiogram showed that there was no flow to the aneurysm, demonstrating successful exclusion of the aneurysm from communication with the ventricle (Fig. 3a). There was also mild mitral valve regurgitation, trivial aortic valve regurgitation, and normal right ventricular and mildly depressed left ventricular function.

The post-operative course was uncomplicated, and the young man was ultimately discharged seven days after surgery. Due to the persistent mild mitral valve regurgitation with mildly depressed left ventricular systolic function, the patient was discharged home on amlodipine for afterload reduction, which subsequently was successfully weaned off prior to systemic CAR-T cell therapy for the relapse. One month post-operatively, his prior aneurysm was no longer seen by echocardiogram, presumably having involuted in the interim (Fig. 3b). No other cardiac complications were detected six months post-operatively, and the most recent echocardiogram demonstrated stable mild mitral valve regurgitation and normal left ventricular systolic function with no evidence of the prior submitral left ventricular aneurysm.

## Discussion

We present the case of an adolescent boy with relapsed B-cell ALL and an incidentally discovered submitral left ventricular aneurysm that was repaired without significant complication. Submitral left ventricular aneurysms are a rare entity in children and are usually believed to be due to congenital weakness of the posterior portion of the mitral fibrous atrioventricular junction, based on reported fetal cases [1–3]. Acquired causes are more commonly reported in the adult population and include tuberculosis, human immunodeficiency virus, myocardial infarction, endocarditis, rheumatic carditis, and Takayasu arteritis [1, 4–7]. The patient presented here had multiple prior echocardiographic studies showing normal intracardiac anatomy, thereby demonstrating a unique case of an acquired submitral aneurysm in a pediatric patient.

Given the prior history of MSSA bacteremia, it is suspected that the patient developed bacterial endocarditis as the catalyst to this aneurysm formation which, though rare, is a known complication. There are two leading theories to describe the mechanism of submitral left ventricular aneurysms in the setting of endocarditis. The first is that an abscess forms at the atrioventricular junction with a subsequent fistulous connection created between the aneurysm and ventricle. The second is that the infection erodes a region of the atrioventricular junction with subsequent disruption and aneurysm formation due to high left ventricular pressures [8, 9]. Since this patient never had echocardiographic evidence of an abscess with echogenic material in the aneurysm, the second hypothesis is the more probable explanation in this case. The MSSA bacteremia was likely partially treated but resulted in weakening of the valve atrioventricular junction at the posterior mitral leaflet. This subsequently led to disruption in the fibrosa of the atrioventricular junction and separation of the left ventricular myocardium from the junction, with high left ventricular pressures causing the aneurysm to form and grow. Fungal bacteremia as the etiology is another consideration especially considering his history of invasive mucormycosis, but this is much rarer as a source of endocarditis.

Patients with a submitral aneurysm can present on a spectrum from asymptomatic (such as in our patient) to sudden death. They most commonly present with heart failure symptoms secondary to mitral regurgitation due to disruption of the fibrous annulus of the posterior leaflet. Alternatively, they may present with symptoms due to impingement of the left circumflex artery and subsequent myocardial ischemia. If cardiac function is compromised, it can be a nidus for thrombus formation, and patients may present with a stroke. Catastrophic rupture may also occur [1, 4]. Data are limited to case reports and small case series regarding the true prevalence and natural history of this disease in children, but given the risks as described above, intervention is preferred [1, 7, 10].

Unique to this patient was the active oncologic disease, which led to significant multidisciplinary discussions about the timing and risks and benefits of proceeding with surgical repair. Among adult patients with history of hematologic malignancy, prior research suggests a similar complication rate and 30-day mortality following cardiac surgery compared to controls, although they are more likely to require post-operative blood product transfusion. Unfortunately, there is scarce data regarding outcomes from cardiac surgery with concurrent hematologic malignancy in the pediatric population [11]. Catheter-based occlusion of LV diverticula has been performed with a satisfactory outcome [12]. However, given the patient’s suspected infectious etiology, there were concerns about tissue integrity. Catheter-based closure, while minimally invasive, was thought to carry undue risk of aneurysm rupture, disruption of the mitral valve apparatus, or embolization of the coils. Ultimately, given the negative bone marrow biopsy indicating localization of the patient’s malignancy to the central nervous system, the lack of bacteremia or fungemia, and adequate blood counts, the decision was made to proceed with surgery during this narrow therapeutic window prior to systemic chemotherapy to allow proper wound healing and minimize post-surgical infectious risks. Surgical repair can be achieved via transatrial or transaneurysmal approach, and the spectrum of surgical treatment spans from patch closure to valvuloplasty to mitral valve replacement depending on the state of the valve at the time of intervention [1, 7, 13].

In conclusion, acquired submitral left ventricular aneurysms are a rare but potentially deadly complication of endocarditis in children. In the setting of comorbidities such as hematologic malignancy, thoughtful multidisciplinary discussion regarding the risks, benefits, timing, and type of intervention is of the utmost importance.

## Data Availability

No datasets were generated or analysed during the current study.
